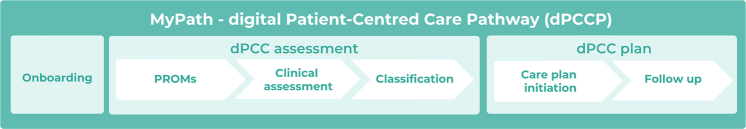# Practical cancer nutrition, from guidelines to clinical practice: a digital solution to patient-centred care

**DOI:** 10.1016/j.esmoop.2025.104529

**Published:** 2025-04-02

**Authors:** K.S. Hustad, L.H. Koteng, A. Urrizola, J. Arends, A. Bye, O. Dajani, L. Deliens, M. Fallon, M.J. Hjermstad, M. Kohlen, G.P. Kurita, T. Lundeby, N. Mitrea, C. Payne, S. Roselló-Keränen, N. Warmbrodt, A. de Wilde, S. Kaasa, J. de Vos-Geelen, B.J.A. Laird, MyPath consortium, K. Absolom, M. Andresen, M. Atter, D. Ausen, S. Bea, K. Beernaert, A. Caraceni, A. Cervantes, K. Cresswell, O. Dajani, J. de Vos-Geelen, L. Deliens, F. Evans, M. Fallon, V. Freitas, V. Fusetti, I. Gonzalez-Barrallo, P. Hall, M.J. Hjermstad, M. Huerta, K.S. Hustad, A. Jacobs, S. Kaasa, L.H. Koteng, G.P. Kurita, H. Larsen, U. Lassen, N.J. Latino, T. Lundeby, E.D. Lundereng, C.C. Lykke, G. Massa, U. Mathiesen, N. Mitrea, D. Mosoiu, S.O. Damink, H. Pappot, K. Pardon, C. Payne, O. Predoiu, A.-L. Scherrens, M. Shkodra, P. Sjøgren, E. Storaas, A. Urrizola, P.H. Utne, F. Van Landschoot, G. Velikova, L. Warrington, N. White, R. Williams

**Affiliations:** 1 Leeds Cancer Centre, St James’s University Hospital, Leeds, UK; 2 Leeds Institute of Medical Research at St James’s, University of Leeds, Leeds, UK; 3 DNV Imatis AS, Porsgrunn, Norway; 4 Institute of Genetics and Cancer, University of Edinburgh, Edinburgh, UK; 5 Department of General Practice and Chronic Care, End-of-Life Care Research Group, Vrije Universiteit Brussel (VUB) & Ghent University, Brussels, Belgium; 6 Fondazione IRCCS Istituto Nazionale dei Tumori, Milan, Italy; 7 Dipartimento di Scienze Cliniche e di Comunità—Dipartimento di eccellenza 2023–2027 Università degli studi di Milano, Milan, Italy; 8 Department of Medical Oncology, INCLIVA Biomedical Research Institute, Valencia, Spain; 9 CIBERONC, Instituto Salud Carlos III, Madrid, Spain; 10 Usher Institute, University of Edinburgh, Edinburgh, UK; 11 European Palliative Care Research Centre (PRC), Department of Oncology, Oslo University Hospital, and Institute of Clinical Medicine, University of Oslo, Oslo, Norway; 12 Department of Internal Medicine, Division of Medical Oncology, GROW—Research Institute for Oncology & Reproduction, Maastricht University Medical Center+, Maastricht, The Netherlands; 13 Department of Scientific & Medical Affairs, European Society for Medical Oncology (ESMO), Lugano, Switzerland; 14 Department of Media and Communication Studies, Vrije Universiteit Brussel (VUB), Imec-SMIT Research Group, Brussels, Belgium; 15 Department of Oncology, Pain and Respiratory Support, Rigshospitalet Copenhagen University Hospital, Copenhagen, Denmark; 16 Department of Anaesthesiology, Pain and Respiratory Support, Rigshospitalet Copenhagen University Hospital, Copenhagen, Denmark; 17 Department of Clinical Medicine, University of Copenhagen, Copenhagen, Denmark; 18 Department of Oncology, Section of Palliative Medicine, Rigshospitalet Copenhagen University Hospital, Copenhagen, Denmark; 19 Department of Oncology, Rigshospitalet Copenhagen University Hospital, Copenhagen, Denmark; 20 Department of Oncology and Palliative Care, North Zealand Hospital, Denmark; 21 Department of Fundamental Disciplines and Clinical Prevention, Faculty of Medicine, University of Transilvania from Brasov, Brasov, Romania; 22 Department of Education and National Development, HOSPICE Casa Sperantei, Brasov, Romania; 23 Department of Medical and Surgical Specialties, Faculty of Medicine, University of Transilvania from Brasov, Romania; 24 Department of Surgery, Maastricht University Medical Centre, Maastricht, The Netherlands; 25 NUTRIM Institute of Nutrition and Translational Research in Metabolism, Maastricht University, Maastricht, The Netherlands; 26 Department of General, Visceral, Vascular and Transplant Surgery, University Hospital Essen, Essen, Germany; 27 European Association for Palliative Care, Vilvoorde, Belgium; 28 Institute for the Study of Science, Technology and Innovation, University of Edinburgh, Edinburgh, UK; 1 European Palliative Care Research Centre (PRC), Department of Oncology, Oslo University Hospital, and Institute of Clinical Medicine, University of Oslo, Oslo, Norway; 2 Department of Medicine I, Medical Centre—University of Freiburg, Faculty of Medicine, University of Freiburg, Freiburg im Breisgau, Germany; 3 End-of-Life Care Research Group, Vrije Universiteit Brussel & Ghent University, Brussels, Belgium; 4 Department of Palliative Medicine, University of Edinburgh, Edinburgh, UK; 5 Department of Dietetics, Maastricht University Medical Centre+, Maastricht, The Netherlands; 6 Department of Clinical Medicine, Faculty of Health and Medical Sciences, University of Copenhagen, Copenhagen, Denmark; 7 Multidisciplinary Pain Centre, Department of Anaesthesiology, Pain and Respiratory Support, Neuroscience Centre, Rigshospitalet—Copenhagen University Hospital, Copenhagen, Denmark; 8 Section of Palliative Medicine, Department of Oncology, Centre for Cancer and Organ Diseases, Rigshospitalet—Copenhagen University Hospital, Copenhagen, Denmark; 9 Department of Fundamental Disciplines and Clinical Prevention, Faculty of Medicine, University of Transilvania from Brasov, Brasov, Romania; 10 Department of Education and Research, HOSPICE Casa Sperantei, Brasov, Romania; 11 European Association for Palliative Care, Vilvoorde, Belgium; 12 Department of Medical Oncology, INCLIVA Biomedical Research Institute, University of Valencia, Valencia, Spain; 13 CIBERONC, Instituto de Salud Carlos III, Madrid, Spain; 14 Section of Clinical Nutrition, Department of Clinical Service, Division of Cancer Medicine, Oslo University Hospital, Oslo, Norway; 15 Department of Surgery, School of Nutrition and Translational Research in Metabolism (NUTRIM), Maastricht University, Maastricht, The Netherlands; 16 Division of Medical Oncology, Department of Internal Medicine, GROW—Research Institute of Oncology & Reproduction, Maastricht University Medical Centre, Maastricht, The Netherlands; 17 Institute of Genetics and Molecular Medicine, University of Edinburgh, Edinburgh, UK; 18 St Columba’s Hospice, Edinburgh, UK

**Keywords:** patient-centred care, nutritional care, nutritional assessment, MyPath, implementation

## Abstract

**Background:**

Malnutrition affects 20%-70% of cancer patients, depending on tumour type, disease stage, and clinical setting. While nutritional care is essential for improving patients’ quality of life and clinical outcomes, it is not systematically integrated into routine cancer care. MyPath is a European Union project aiming to implement patient-centred care (PCC) at nine European cancer centres using implementation science. Multidisciplinary teams have developed standardised digitally supported PCC pathways based on patient-reported outcomes (PROs) with linked evidence-based management options. Through systematic assessment and management of common symptoms and psychosocial problems in cancer patients, MyPath aims to facilitate changes in clinical practice to improve PCC for all. As part of this, the MyPath Nutrition Care Pathway (NCP) aims to facilitate necessary clinical changes to routinely assess and address nutrition in all patients.

**Materials and methods:**

Between September 2022 and August 2024, an international multidisciplinary team reviewed evidence-based nutrition guidelines to select relevant PROs and other variables necessary to systematically assess patients, allowing for tailored nutritional care.

**Results:**

The MyPath NCP assessment relies on nutritional status (Malnutrition Screening Tool for malnutrition risk, modified Global Leadership Initiative on Malnutrition criteria for malnutrition, and body mass index/weight change for obesity/unintentional weight gain), health status (functional status, cancer diagnosis and prognosis, and prehabilitation needs), and inflammatory status (C-reactive protein levels). Based on this assessment, the digital solution suggests tailored, evidence-based nutritional interventions. Continuous monitoring through PROs and clinical consultations will customise care to patients’ dynamic nutritional needs. The first version of this digital solution will be piloted in 2025.

**Conclusions:**

Inconsistent implementation of nutrition guidelines is a key challenge in cancer care. The MyPath NCP offers an accessible, patient-centred assessment and management system that integrates nutritional care into routine cancer care, providing a versatile solution that can be implemented across diverse health care settings.

## Highlights

• MyPath aims to implement PCC at nine European Union cancer centres.
• The MyPath NCP is developed to integrate nutritional care into routine cancer care.
• The MyPath NCP classifies patients based on nutritional, health, and inflammatory status, through systematic assessment.
• The MyPath digital solution provides tailored, guideline-based care options.
• The first version of the MyPath digital solution will be piloted in 2025.

## Introduction

Launched in 2021, Europe’s Beating Cancer Plan aims to reduce cancer burden for patients, their families, and health systems.^1^ It emphasises patient-centred and nutritional care as key components, receiving broad endorsement.^2^ While the adoption of these principles varies internationally, they are widely accepted and supported by extensive evidence.^3^

Optimising nutrition should be regarded a key aspect of cancer treatment for several reasons.^4^ Research has demonstrated that improving nutritional status positively influences clinical outcomes. This includes improvement in post-surgery recovery, survival rates, tolerance of anticancer therapy, and quality of life, both in curative and non-curative cohorts.^5^, ^6^, ^7^, ^8^ There are also clear cost-saving benefits associated with improved nutritional status, such as shorter hospital stays and less need for contact with health care professionals (HCPs).^9^^,^^10^ Patient-centred nutritional care is also emerging as a human right^11^ and should therefore be an integral component of health care. Finally, the abundance of data, which exist and have been formulated into guidelines for addressing nutrition in cancer care, shows that nutritional cancer care is firmly evidence-based.^12^, ^13^, ^14^, ^15^, ^16^

Despite this, nutritional care remains under the radar in routine oncology consultations, often due to limited availability of resources, lack of training, and difficulties with applying classification systems and guidelines.^7^^,^^17^, ^18^, ^19^ Further, even though evidence-based guidelines advise that optimising nutrition improves outcomes, these are often not used in day-to-day clinical practice and concerns exist about their feasibility. Time constraints are also a common barrier.^20^

Aligned with the oncology paradigm of ‘staging the tumour, treating the tumour’, nutritional care should ‘stage the patient’ and subsequently ‘treat the patient’. Staging the patient is done to some extent through performance status classification. However, staging in terms of nutritional and even inflammatory status has been shown to predict survival, improvements in quality of life, and response to cancer therapy.^21^, ^22^, ^23^ This is advocated by nutrition guidelines^14^; thus, clear principles exist to ‘treat the patient’. Nutrition guidelines can help support this if they can be adopted easily and systematically into routine clinical practice.

Research shows that standard cancer care can significantly benefit from greater active involvement of cancer patients in decisions about their own care, an approach known as patient-centred care (PCC). PCC focuses on the patient ‘living with the disease’, and one key element is systematically asking patients about their symptoms and function. MyPath is an implementation project funded by the European Union and managed by a European collaborative of 15 partners.^24^ MyPath aims to establish integrated and efficient PCC that is respectful of, and responsive to, individual patient preferences, needs, and values. With this aim, the project is developing a digital solution based on digital PCC pathways, custom-made for each individual patient, which will include real-time communication of symptoms and care preferences using electronic patient-reported outcome measures (PROMs). MyPath will translate the proven concept of PCC into practical reality, in cancer care.

The aim of this study is to describe the development of a cancer nutrition care pathway (NCP) that integrates evidence-based nutrition guidelines into the digital MyPath solution. Our hypothesis is that an NCP, combining evidence-based guidelines, can be implemented into specialist cancer centres alongside routine clinical care.

## Materials and methods

### MyPath meta-architecture

The MyPath project was initiated in 2022 and consists of three periods: the design phase (September 2022-August 2024), the implementation phase, during which the solution will be implemented in nine European cancer centres with iterations of content and implementation strategy (September 2024-August 2026), and the evaluation phase (September 2026-August 2027). During the design phase, the meta-architecture of MyPath was developed and the components were incorporated as illustrated in Supplementary Figure S1, available at https://doi.org/10.1016/j.esmoop.2025.104529.

Firstly, patients are onboarded to the MyPath digital solution and before a clinical consultation, patients complete PROMs using the digital tool ‘MyPath Patient’, which is based on a system called Eir.^25^ This assessment can be carried out either at home or at hospital through a mobile phone application or web page. Before meeting the patient, the clinician will review the PROMs in the clinician part of the tool, ‘MyPath Clinician’. The digital solution will guide the clinician through a clinical consultation, addressing the patients’ needs and care priorities. The information from the PROMs and the clinical consultation, will lead to a diagnosis and patients will be classified into different subgroups. The clinician will tailor PCC plans based on their needs and preferences. These PCC plans are multidisciplinary and evidence-based that include specific care options. Follow-up can be either digital or a physical clinical consultation when needed. The meta-architecture is further described in ‘MyPath: the roadmap to implement patient-centred care’.^26^

### Development of the MyPath nutrition care pathway

#### Multidisciplinary steering group

In February 2023, an international multidisciplinary steering group (Supplementary Table S1, available at https://doi.org/10.1016/j.esmoop.2025.104529) was created to develop the nutrition content in MyPath—the MyPath NCP. Participants included clinical dietitians, nutritionists, medical oncologists, palliative care experts, nurses, and researchers from six European countries. Group members included those aligned with the European Society for Clinical Nutrition and Metabolism (ESPEN),^12^ the Society on Sarcopenia, Cachexia and Wasting Disorders (SCWD), and the European Society for Medical Oncology (ESMO).^13^ A coordinator and a scientific lead were appointed to provide the necessary support regarding the development process, and to facilitate the group discussions ensuring the use of evidence-based resources. The group held a total of 17 meetings until September 2024 in addition to local follow-up meetings attended by group representatives.

The remit was to develop a cancer NCP based on evidence-based nutrition guidelines. This included developing an assessment and management system, with care options tailored to the individual patient, cancer type, stage of disease, and therapeutic strategy, i.e. (neo)adjuvant, life-prolonging cancer therapy, or palliative care only. Important steps in the design of the nutritional assessment were to evaluate existing PROMs, refine and add clinical algorithms to define patient categories and classifications according to a decision-tree model, and connect the patient to a personalised care plan.

#### Review and appraisal process

The NCP development process involved different rounds. The first round consisted of a detailed review and appraisal of relevant nutrition guidelines.^12^, ^13^, ^14^, ^15^, ^16^ As group members are aligned with ESPEN, ESMO, and SCWD, they were familiar with key reference material from these societies. Subsequently, existing guidelines and screening tools were ratified and aligned with the exploration of existing clinical practice across various European cancer centres, expert opinions, and clinical experience, with the aim of making them more applicable in clinical practice.

#### Content selection

The following round involved deciding which content was collected in the PROM retrieval, and thus made available before the clinical consultation, and which would be provided by the clinician based on the clinical consultation and baseline information available in the MyPath solution from the electronic health records (EHRs), if needed. Content selection depended on choosing items that held prognostic and clinical relevance, specifically for oncology-based nutritional outcomes, as well as feasibility.

Work from the NCP steering group was reported back to the MyPath management group and external stakeholders, which included clinicians actively engaged in the care of patients with cancer and information and communication technology developers. This collaborative and multifaceted approach ensured a comprehensive development of the NCP, incorporating diverse perspectives, guidelines, and expertise from both internal and external stakeholders, and matched with the unique requirements of the oncology population.

## Results

### MyPath NCP assessment

**Figure 1:**
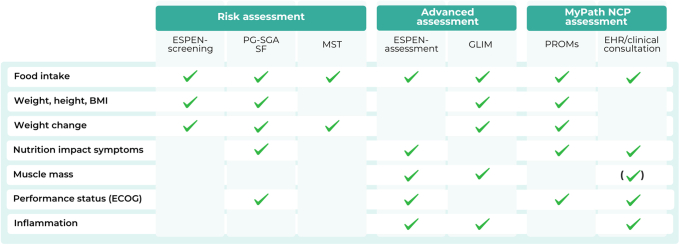
**International nutritional assessment recommendations.** Key tenets of the MyPath NCP assessment compared with recommendations in international guidelines and screening tools.^12^, ^13^, ^14^, ^15^, ^16^ The phenotypic criterion for low muscle mass is not included by default as this would require a separate measurement not routinely carried out at most sites; however, it may be included in sites where this is measured routinely. BMI, body mass index; ECOG, Eastern Cooperative Oncology Group; EHR, electronic health record; ESPEN, European Society for Clinical Nutrition and Metabolism; GLIM, Global Leadership Initiative on Malnutrition; MST, Malnutrition Screening Tool; NCP, nutrition care pathway; PG-SGA SF, Patient-Generated Subjective Global Assessment Short Form; PROM, patient-reported outcome measure.

The items that were selected for assessment of nutritional status were PROMs [nutrition impact symptoms (NISs), dietary intake], biological data (e.g. biomarkers of inflammation),^27^ clinical data [weight, degree of weight loss, body mass index (BMI)],^28^ and functional status [self-reported,^29^ based on the Eastern Cooperative Oncology Group performance status (ECOG-PS)].^22^ Figure 1 summarises the key tenets of the MyPath NCP assessment, comparing them with recommendations for nutritional risk assessment, advanced nutritional assessment, and components of frequently used screening tools in cancer patients.^12^, ^13^, ^14^, ^15^, ^16^^,^^29^

**Figure 2:**
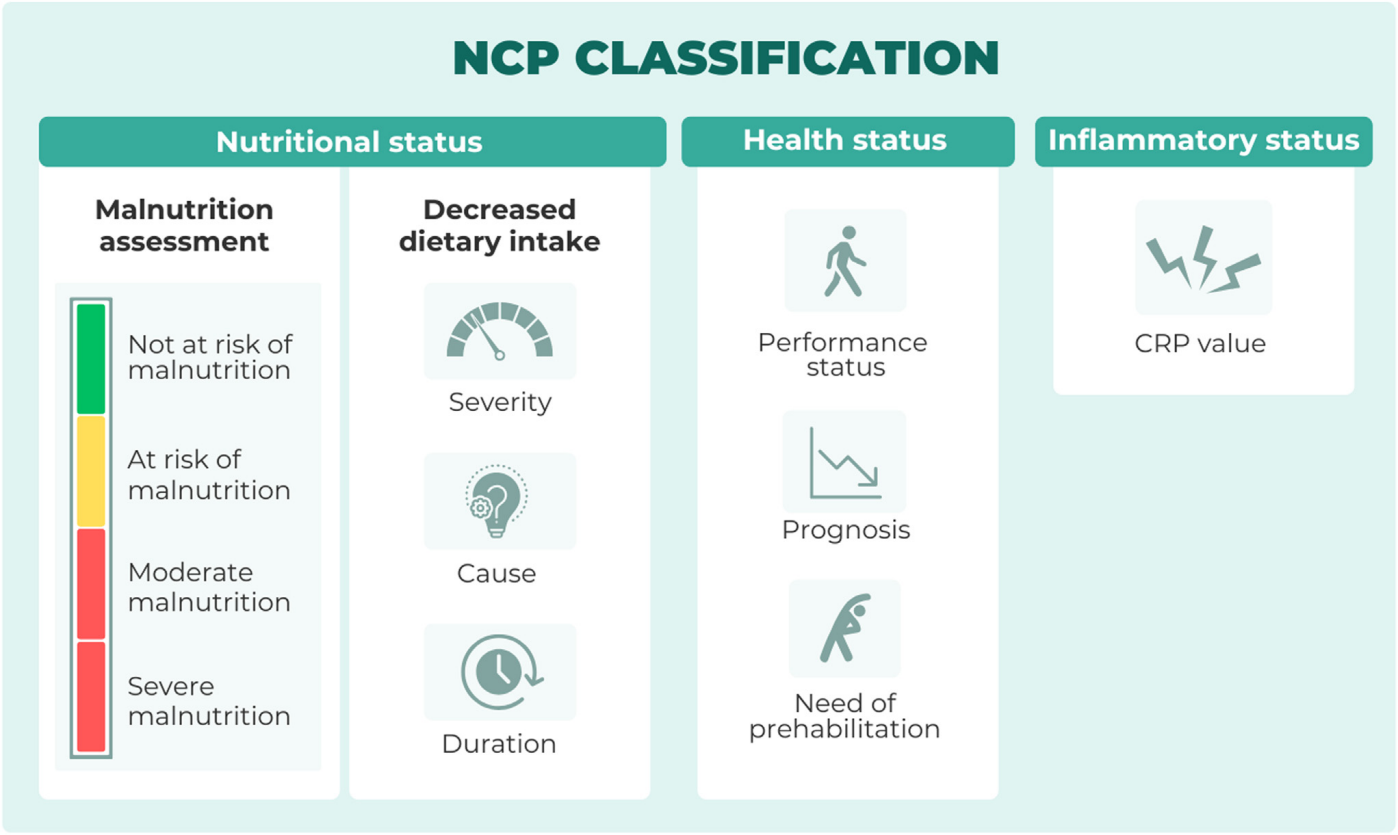
**NCP classification.** In the MyPath NCP, classification relies on three central pillars: nutritional status including malnutrition and dietary intake assessment, health status, and inflammatory status. CRP, C-reactive protein; NCP, nutrition care pathway.

Various combinations of the final variables led to the agreement of three central aspects of the NCP classification—nutritional, health, and inflammatory status (Figure 2). Reliant on this classification, the digital solution provides clinical care options, which when combined with shared decision making (between patient and HCP) leads to initiation of management tailored to the patient’s needs. The results of this process follow along with an exemplar case showing how the NCP is personalised to individual patients.

### Nutritional status

#### Malnutrition assessment

**Figure 3:**
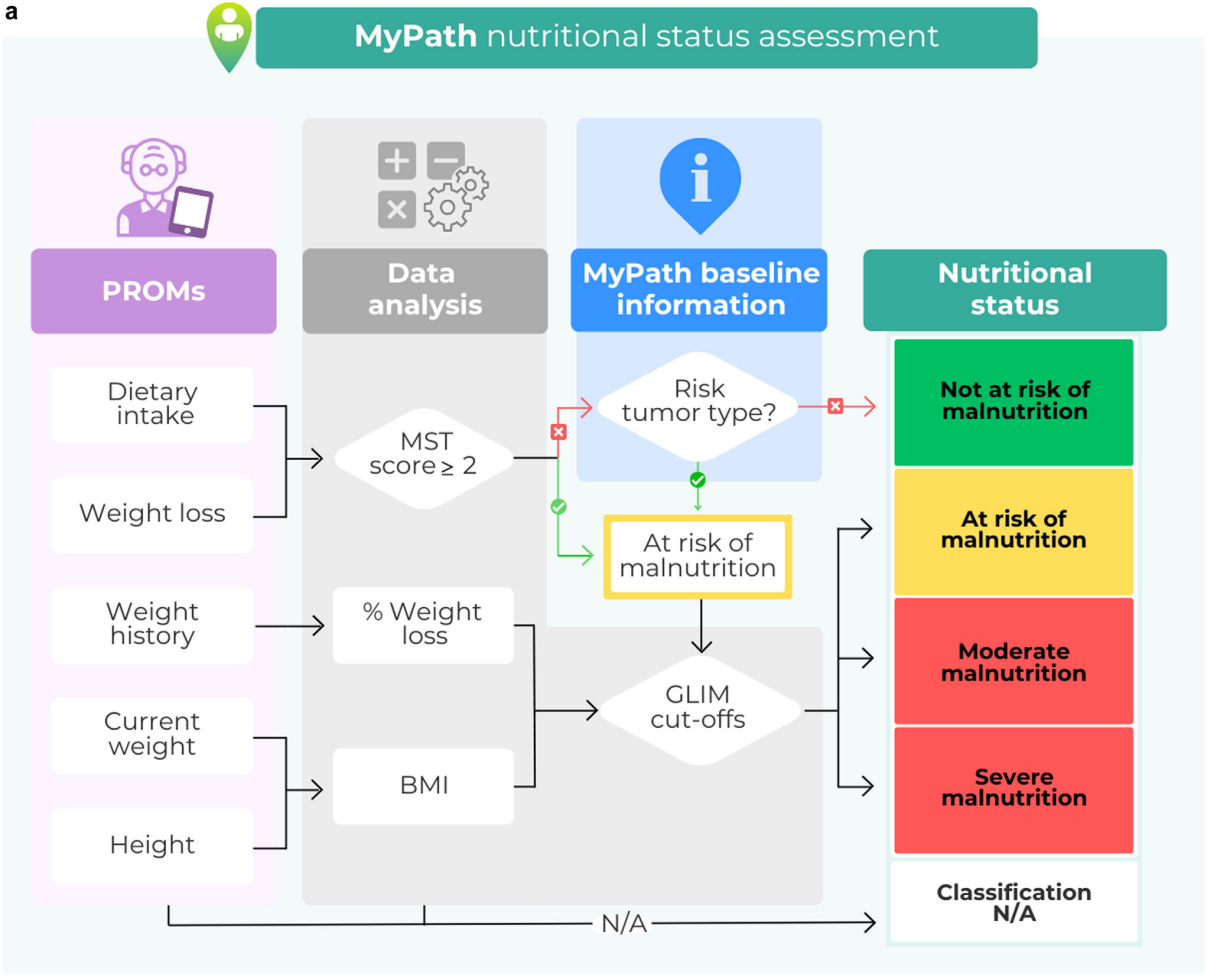

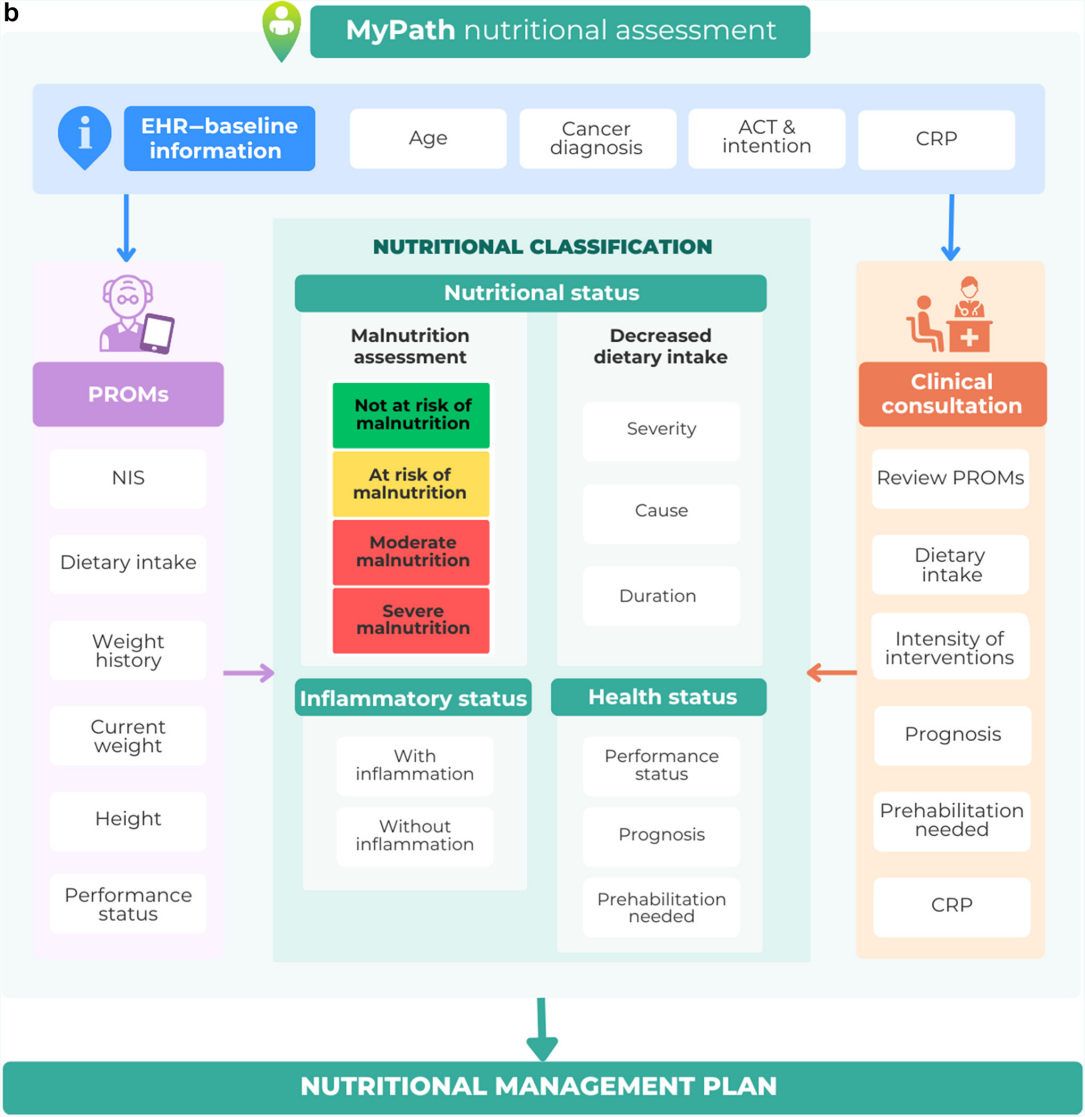
**MyPath nutritional assessment.** (A) MyPath nutritional status assessment. Using the MyPath NCP classification system, the patients will fill in PROMs, and inputted data will be analysed. Patients are classified as at risk of malnutrition if they have an MST score ≥2 or if they have a ‘risk tumour type’ (i.e. cancer diagnosis associated with high prevalence of malnutrition). Patients are then classified as having moderate or severe malnutrition based on the GLIM cut-offs for weight loss and BMI. (B) Summary of the MyPath NCP assessment. A combination of information provided by the patient via PROM retrieval, baseline data available within the MyPath solution and the electronic health records, and information retrieved during the clinical consultation by the clinician provides the main pillars of the classification: nutritional, health, and inflammatory status of the patient. This will lead to the initiation of the patient-specific nutritional management plan. ACT, anticancer treatment; BMI, body mass index; CRP, C-reactive protein; GLIM, Global Leadership Initiative on Malnutrition; MST, Malnutrition Screening Tool; N/A, not available; NIS, nutrition impact symptom; PROM, patient-reported outcome measure.

The algorithm used to assess malnutrition is shown in Figure 3A. The Malnutrition Screening Tool (MST) was chosen as it has recently demonstrated validity in adult populations across health care settings^30^ and in ambulatory cancer patients specifically.^31^ Additionally, MST consists of only two questions that can be self-reported. Using the MST scoring criteria, patients are classified as ‘not at risk of malnutrition’ (MST score 0 or 1) or ‘at risk of malnutrition’ (MST score ≥2).^32^

The exact wording of the MST items can be adapted when needed, and/or include help text, to ensure the patients’ understanding. Patients with an MST score of 0 or 1 may be considered at risk if they have a cancer diagnosis highly associated with malnutrition (e.g. lung or pancreatic cancer) and/or the treating physician considers that the planned cancer treatment will compromise the patient’s nutritional or health status.

Patients are assessed for malnutrition (moderate or severe) based on the Global Leadership Initiative on Malnutrition (GLIM) phenotypic criteria cut-offs for non-volitional weight loss and/or low BMI.^33^ In those cases where there are insufficient data, a classification will not be available. The phenotypic criterion for low muscle mass is not included by default as this would require a separate measurement not routinely carried out at most centres; however, it may be included in centres where this is measured routinely.

In addition to (risk of) malnutrition, the solution will be able to identify patients experiencing unintentional weight gain during or after treatment and suggest tailored nutritional management accordingly.

#### Decreased dietary intake

Evaluation of dietary intake stands as a pivotal component within the context of a comprehensive nutritional assessment. Thus, the NCP assesses the presence of decreased dietary intake, and when present—the cause, severity, and duration.

### Health status

To tailor the intervention(s) to patients’ needs and preferences, assessment of the health status of the patient is required. This includes performance status (both patient-reported and clinician-reported), cancer diagnosis, current anticancer treatment and intent, and the need for prehabilitation (e.g. patient with borderline resectable pancreatic cancer who will undergo surgery). Subsequently, the intensity, complexity, and compliance requirements of the nutrition management plan are aligned with, and responsive to, the patient’s health status.

### Inflammatory status

C-reactive protein values distinguish between malnourished patients with, and without, inflammation, using 10 mg/dl as a cut-off.^21^^,^^34^ If the presence of infection is suspected, CRP may need to be assessed on two separate occasions to ensure it is raised. The presence of systemic inflammation and acute weight loss, in the presence of loss of muscle (sarcopenia), will help differentiate cancer-related sarcopenia from non-cancer causes, which usually develop insidiously and are due to anabolic resistance, inactivity, or chronic disease (incorporated into the routine clinical consultation), with less inflammation.

### Data retrieval

Data used for nutritional assessment are collected from PROM retrieval, baseline data available in the MyPath digital solution (e.g. age, cancer diagnosis), and the clinical consultation. The baseline data are either manually registered or retrieved automatically when the solution has a high integration with the EHR. A summary of the MyPath NCP is provided in Figure 3B.

#### PROM retrieval

Before each clinical consultation, patients complete the nutrition risk assessment which includes their weight history (current weight, perceived weight loss), dietary intake (presence of decreased intake and severity), and physical function (box 4 ‘activities and function’ in Patient-Generated Subjective Global Assessment Short Form, based on ECOG-PS). The MyPath solution will then calculate the BMI and percentage of weight change. For first-time users with no prior data, patients will provide self-recalled weight from 6 and 12 months previously. Based on these items, the system can provide a preliminary assessment of the patients’ nutritional status.

In addition to these items, patients will report presence of NISs (nausea, vomiting, diarrhoea, constipation, lack of appetite, dry mouth, mouth sores, altered sense of taste, altered sense of smell, feeling full quickly, problems swallowing, psychological distress, dyspnoea, pain), indicating the intensity of symptoms using a numerical rating scale from 0 to 10 (0 = absence of the symptom and 10 = worst intensity imaginable for that symptom), and additional information when required. This symptom assessment is based on the Common Terminology Criteria for Adverse Events (v.4.0 and 5.0).

#### Clinical consultation by HCP

During the clinical consultation, the HCP will confirm the information provided by the patient and continue the nutritional assessment, also evaluating if there is potentially hidden weight loss (e.g. due to ascites, pleural effusions). Further, the HCP will assess inflammation, the need for prehabilitation, and cause and duration of decreased dietary intake, if reported. For patients at risk of malnutrition, or with malnutrition, the intensity and invasiveness of the nutritional interventions will be considered based on the health status (performance status, cancer diagnosis and prognosis, and prehabilitation needs).

Using adaptive questioning, the system will ask clinicians to input additional information, when needed, tailored to prior answers or the preliminary classification. We anticipate that the time taken to carry out the NCP assessment will be between 10 and 15 min.

### Nutrition care pathway—care options

Based on the nutritional assessment, the digital solution will suggest nutritional care options tailored to the patient’s needs. The HCP then discusses these with the patient and, based upon shared decision making, indicates which ones are initiated.

By using a stepwise approach to nutritional care, the least resource-intensive and most effective intervention to meet the patients’ needs is selected, whereby focusing on the underlying reason for nutritional support is key. Addressing underlying issues such as nausea, pain, and swallowing difficulties may be essential for successful nutritional interventions. For some patients, simply reducing or eliminating these barriers can increase food intake effectively, while for others, it may be necessary to start with more invasive interventions such as enteral or parenteral nutrition at an early stage.

All patients may receive general information on the importance of physical activity and diet during and after cancer diagnosis, together with recommendations for physical activity. Additionally, the solution will provide dietary advice based on nutritional status, suggestions on when to initiate medical nutrition therapy (oral nutrition supplements including patient instructions for use, enteral nutrition/tube feeding, parenteral nutrition), care options for management of NISs including dietary advice, and diagnosis- or treatment-specific care options such as dietary advice specific for prehabilitation and instructions for use of pancreatic enzyme replacement therapy.

**Figure 4:**
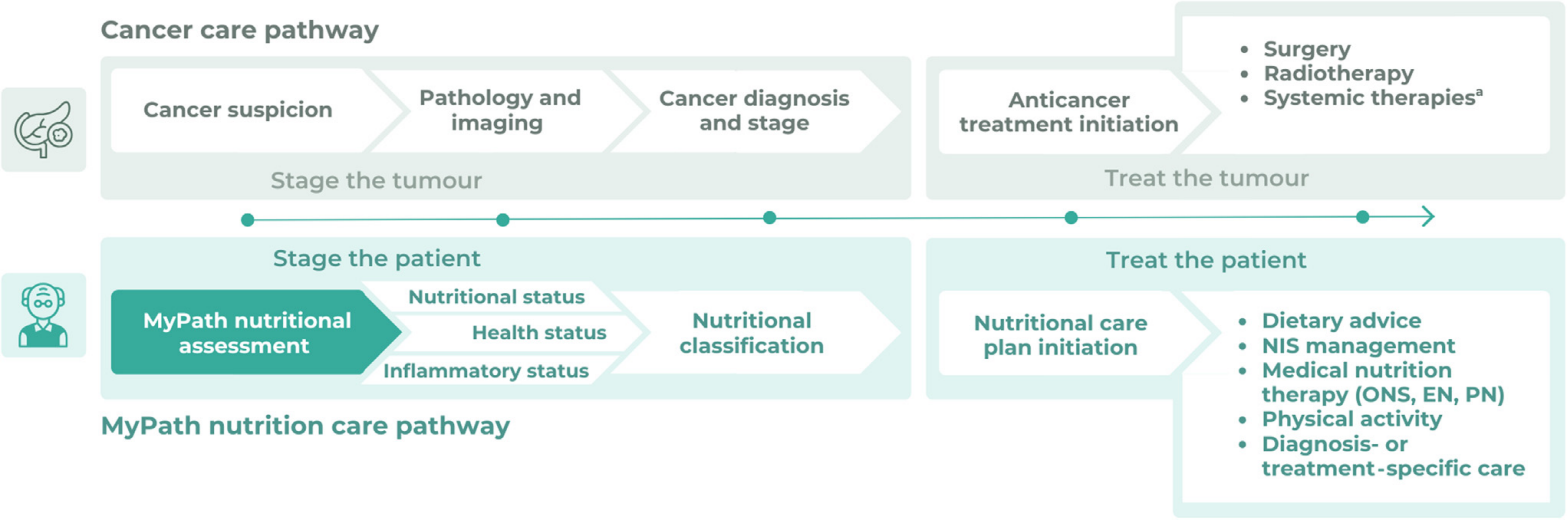
**A two-step approach for managing nutrition in patients with cancer in parallel with cancer care.** Nutritional assessment occurs simultaneously with cancer staging and care, irrespective of the patient’s position in the disease trajectory, and leads to the initiation of a nutritional care plan tailored to patient’s needs. EN, enteral nutrition; NIS, nutrition impact symptom; ONS, oral nutrition supplement; PN, parenteral nutrition. ^a^Include chemotherapy, immunotherapy, targeted therapy, and others.

In practice, the digital solution will take patients from nutritional assessment to management in parallel with their oncological care (Figure 4).

MyPath care options are categorised into patient educational resources, self-management advice, non-pharmacological interventions, pharmacological interventions, diagnostic procedures, and referrals to other clinicians.

Continuous monitoring via PROM retrieval, and subsequent reappraisal, with clinical consultations when needed, will assess compliance and efficacy of the initiated measures, and consequently update the classification. Further, the care options will be adapted to the current nutritional, health, and inflammatory status of the patient.

### Stakeholder engagement and local adaptations

To enhance patient and clinician engagement, HCPs and patients were actively involved in the development of the MyPath digital solution through iterative processes during the lifetime of the project.^24^ Moreover, hospitals where MyPath will be introduced are being studied a priori through an implementation science model so that it will adapt to local practices, guidelines, and resources. The first version of this digital solution will be piloted in 2025.

### Exemplar case

**Figure 5:**
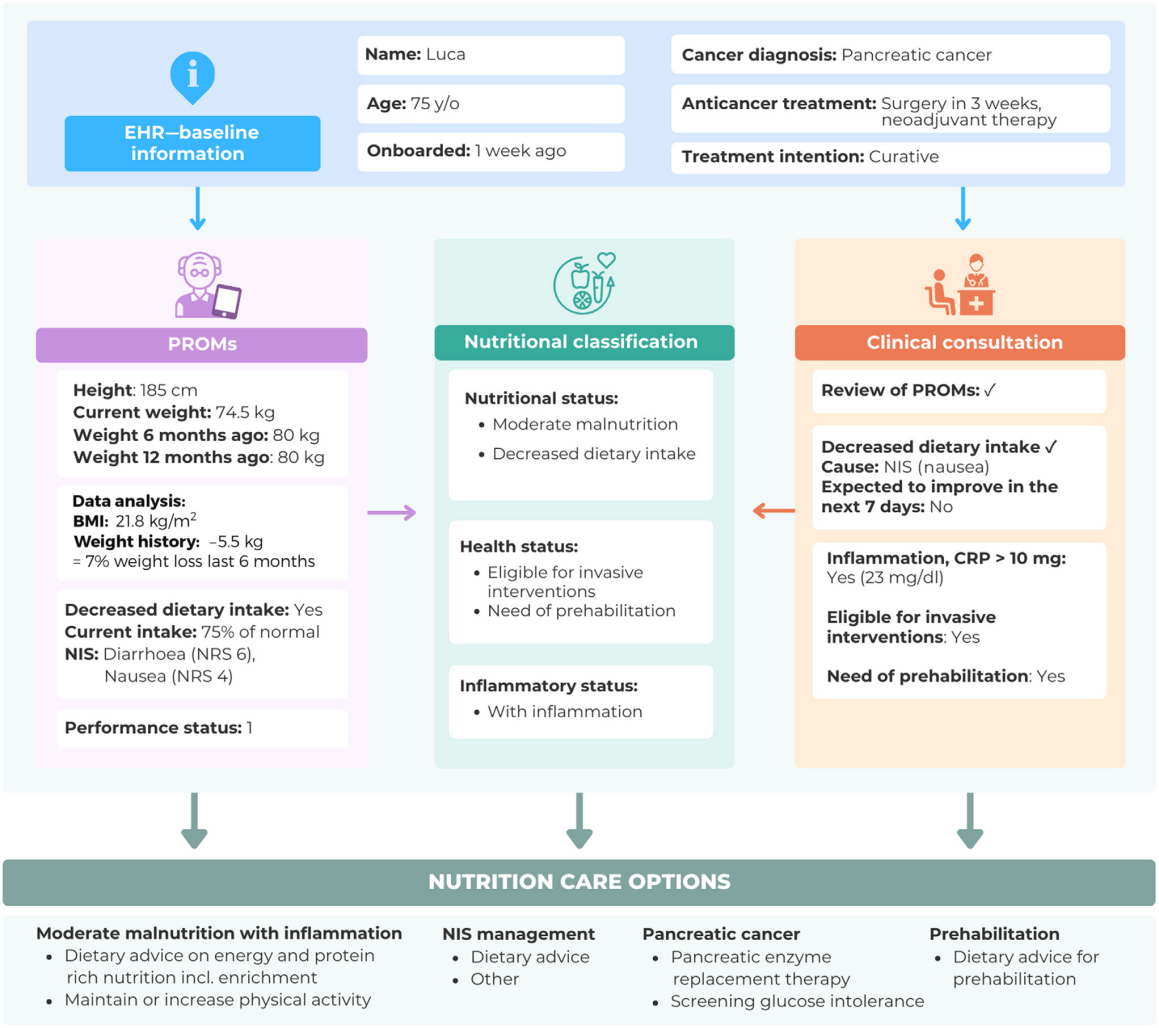
**MyPath NCP in practice—exemplar case.** BMI, body mass index; CRP, C-reactive protein; NCP, nutrition care pathway; NIS, nutrition impact symptom; NRS, numerical rating scale.

Figure 5 includes an exemplar case to illustrate how the MyPath NCP will work in practice and how assessment and management are adapted to the individual patient with cancer.

## Discussion

A prevailing challenge in addressing nutrition during cancer care lies in the sporadic implementation of existing guidelines within routine clinical practices. Recognising this critical gap, our innovative approach, the MyPath NCP, seeks to address this deficiency by proposing a more accessible ‘easy-to-use’ patient-centred assessment and management pathway(s). This is based on both international and local nutrition guidelines and is the first NCP that takes guidelines and evidence into routine clinical practice through a patient-centred, digital solution.

### The complex interplay between nutrition and cancer treatment

In elucidating the complex interplay between nutrition, the patient, and cancer treatment, it becomes evident that underlying nutritional status and metabolic changes will affect the cancer and patient outcomes.^27^ The multifaceted relationship between nutritional status and cancer development is supported by a growing body of research that highlights that specific interventions targeting nutrition, delivered at the right time alongside optimal tumoricidal therapy, may promote optimal outcomes.^5^^,^^35^, ^36^, ^37^ Unravelling these intricate connections is of paramount importance for supporting the patient, while also treating the tumour. While the nuances of nutrition in cancer are clear, it is imperative to acknowledge the dynamic nature of this relationship. In addition, the diversity of cancer types, individual variability, and the intricate molecular mechanisms that underlie the influence of dietary factors on treatment response, patient well-being, and nutritional parameters (e.g. appetite, inflammatory biomarkers) need to be considered. Moreover, while knowledge to improve nutritional outcomes in cancer exists,^12^^,^^13^ there are multiple barriers to initiating nutritional interventions in clinical practice.^19^^,^^38^^,^^39^ Through the MyPath NCP, we are now in the position to catapult this knowledge into clinical and patient care.

### A structured assessment and management framework

In pursuit of a more comprehensive and accessible approach to addressing the nutritional needs of patients with cancer and cancer survivors, we advocate for the implementation of a stepwise and user-friendly NCP as part of the MyPath digital solution. This aims to integrate seamlessly into routine clinical practice, offering a structured assessment and classification framework. The framework tailors nutritional care options to the specific nutritional needs identified or anticipated, throughout the whole cancer trajectory. This synergistic integration of nutrition into the broader spectrum of cancer management marks a significant stride towards fostering holistic well-being in patients grappling with the complexities of a cancer diagnosis and treatment, and also enabling oncology treatments to have the best chance of success.

The first step in this process is assessment. Although multiple, nutritional classifications exist (e.g. BMI/degree of weight loss, modified Glasgow Prognostic Score, GLIM criteria^28^^,^^33^^,^^40^) with some of these included in highly regarded guidelines, there is currently a notable absence of their routine use in clinical practice for patients with cancer. The unsuccessful widespread implementation in routine clinical practice, despite ongoing efforts, illustrates the unique and complex nature of nutritional needs within the oncology population, but also potentially a didactic need to plant nutritional care within the core components of good oncology care.^39^ The imperative within this lies in effectively identifying those patients who require timely intervention and delivering tailored nutritional care accordingly, in a pragmatic and simple way.

In addressing this imperative, the MyPath NCP prioritises practicality and actionability, steering away from the sole pursuit of a scientific diagnosis. The system can accurately identify patients in need of targeted management, ensuring that subsequent nutritional care is not only effective but also seamlessly integrated into the broader spectrum of cancer treatment,^41^ acknowledging the dynamic and varied nature of nutritional requirements in the oncology setting. Moreover, the proposed nutritional assessment system is designed to be accessible without requiring specific technology or procedures. This ensures applicability across different health care centres and for various clinicians, promoting its widespread use.

### Benefits and features

The MyPath solution introduces several benefits, such as digital support that retrieves PROMs and makes them available to the HCP. In contrast with standalone applications, MyPath as a complete and comprehensive solution also guides clinicians in efficiently gathering necessary information and automatically provides the assessment and corresponding management algorithms. Thus, MyPath aims to support the clinical processes needed for the implementation of PCC. The emphasis on ease of use is pivotal in ensuring that clinicians, regardless of their geographic location or clinical setting, can readily incorporate the assessment and management pathways provided by MyPath into their routine practice. This will not only streamline the process of nutritional assessment but also facilitate the implementation of tailored interventions, thereby improving the overall quality of cancer care on a global scale. Furthermore, MyPath ensures that individuals undergoing cancer care become active participants in their nutritional assessment and management. This empowers patients and fosters a collaborative approach between HCPs and those receiving care.

### Strengths and limitations

The strengths of MyPath include its novelty, uniqueness, and necessity as an instrument, offering an opportunity for enhancing the quality of life for patients with cancer across Europe. Its simplicity ensures usability by any clinician, leveraging digital PROMs for a customised approach tailored to individual patients throughout the course of cancer therapy and beyond. MyPath aims for applicability in diverse locations, settings, and types of services, with the overarching goal of disseminating high-quality support uniformly throughout Europe. In development of the NCP, the biggest strength is the collaborative effort of clinicians and researchers, ensuring that the pathway is built upon the most recent guidelines and research. This results in a comprehensive and effective tool for nutritional support. Furthermore, the rigorous discussions and careful decision making during the development phase led to a streamlined pathway, balancing simplicity and functionality for practical use in clinical settings.

Despite its strengths, the MyPath NCP is not without limitations. While it functions effectively in the current landscape, further adaptations may be necessary for integration into different clinical centres following testing with patients. We acknowledge that the selection of only nine cancer centres may introduce potential bias, as these centres may not fully represent the diversity of health care facilities providing cancer care. The MyPath study, and the NCP within it, is a first step but implementation of the NCP in more general settings, such as community hospitals, requires further exploration. Future studies should consider a broader range of health care settings to better understand the adaptability and scalability of the NCP across different types of institutions. Another potential challenge is assessing how the NCP integrates with existing local guidelines. However, MyPath NCP is designed to be configurable, enabling different sites to incorporate additional modules as needed. Additionally, potential resistance to change and limitations in resource availability pose challenges. The flexibility of the solution ensures adaptability to various implementation barriers and challenges across different health care settings. Time constraints highlight the necessity for the integration and crosstalk between MyPath and EHRs to avoid duplication of effort. This has been identified as a priority but yet not attained.

### MyPath generalisability and local configuration

The MyPath NCP will be further adapted for use at different centres, matching local practice, guidelines, and available resources (e.g. some centres have the potential to include muscle mass assessment routinely for all patients with cancer). In addition to the local configuration, during the implementation phase, feedback and data collected from HCPs, patients, and caregivers can further modify or tailor the assessment to ensure its applicability in clinical practice.

Crucial to the success of MyPath is a unique agile methodology that embraces the principles of applied research and user-led development.^24^ The development process of MyPath involves integration with existing health care systems and it is being tested, modified, and re-tested to ensure real-world applicability. The involvement of both internal and external stakeholders is at the core of developing care pathways that align with existing guidelines, but also allow appropriate modification to ensure they are practical in each clinical setting. These overarching principles have been applied to the development of all care pathways.

Supplementary Table S2, available at https://doi.org/10.1016/j.esmoop.2025.104529, details the location, health care setting, and tumour type being studied. MyPath will be implemented at nine locations across Europe, including cancer centres and palliative care units. At the cancer centres, tumour types include hepato-pancreato-biliary cancer, metastatic breast and prostate cancer, and advanced lung cancer.

### Next steps: testing and implementation across European cancer centres

The MyPath NCP, as part of the larger MyPath study, is being developed with stakeholders and will then be tested in real-world clinical settings before full implementation across the nine centres. This comprises two key phases: firstly, understanding clinical contexts through qualitative research, including staff interviews and observations, to identify barriers, facilitators, and infrastructural needs and secondly, conducting pilot trials in selected centres, starting in Oslo, to refine MyPath based on integration with existing care practices. The main trial will recruit 20 000 patients, with in-depth data collection from three sites and validation across six other sites. The NCP will be assessed as part of this. A more detailed description of MyPath is available.^24^^,^^26^^,^^42^

### Conclusion

Inconsistent implementation of nutrition guidelines is a key challenge in cancer care. The MyPath NCP offers an accessible, patient-centred assessment and management system that seamlessly integrates nutritional care into routine cancer care, providing a versatile solution that can be implemented across diverse health care settings.

## Figures and Tables

**Supplementary Figure 1:**