# Supplementary material for: Practical cancer nutrition, from guidelines to clinical practice: a digital solution to patient-centred care
Source: ESMO Open. 2025 Apr 2;10(4):104529. doi: 10.1016/j.esmoop.2025.104529 (PMC11998113; doi:10.1016/j.esmoop.2025.104529)
Supplement: Supplementary Table 1 [file mmc1.docx]

**Expert group in pathway development**

| **Name** | **Country** | **Profession** | **Work location** | **International affiliations** |
| --- | --- | --- | --- | --- |
| Judith de Vos-Geelen (scientific lead) | The Netherlands | MD, PhD. Medical oncology. | Hospital, university | ESMO |
| Maxime Kohlen | The Netherlands | Dietitian. | Hospital, university |  |
| Anouk de Wilde | The Netherlands | MD, PhD student. | Hospital, university |  |
| Barry Laird | UK | Professor. MD Palliative medicine. | Hospital, University | ESMO, SCWD |
| Susana Roselló Keränen | Spain | MD, PhD. Medical oncology. | Hospital | ESMO |
| Nicoleta Mitrea | Romania | Advanced practice registered nurse. PhD. Palliative care. | Hospital |  |
| Jann Arends | Germany | MD Cancer cachexia. External volunteer. | University | ESPEN |
| Kristin Solheim Hustad (coordinator) | Norway | Clinical dietitian PhD. Researcher. | Hospital |  |
| Stein Kaasa | Norway | Professor. MD Palliative medicine. | Hospital, University | ESMO |
| Lisa Heide Koteng | Norway | Clinical dietitian. | Hospital |  |
| Amaia Urrizola | Norway | MD, PhD. Post doc. | Hospital |  |
| Olav Dajani | Norway | MD, PhD. Medical oncology. | Hospital |  |
| Asta Bye | Norway | Professor. Clinical dietitian PhD. | Hospital |  |
| Nicole Warmbrodt | Norway | Clinical dietitian. | Hospital |  |
| ESMO; European Society for Medical Oncology, SCWD; Society on Sarcopenia, Cachexia, and Wasting Disorders, ESPEN; European Society for Clinical Nutrition and Metabolism | | | | |
