# Supplementary material for: Practical cancer nutrition, from guidelines to clinical practice: a digital solution to patient-centred care
Source: ESMO Open. 2025 Apr 2;10(4):104529. doi: 10.1016/j.esmoop.2025.104529 (PMC11998113; doi:10.1016/j.esmoop.2025.104529)
Supplement: Supplementary Table 2 [file mmc2.docx]

| **Study centre** | **Country** | **Setting** | **Tumour type(s)** |
| --- | --- | --- | --- |
| Oslo University Hospital | Norway | University Hospital/Cancer centre | Pancreatic cancer |
| Instituto de Investigación Sanitaria | Spain | University Hospital/Cancer centre | Pancreatic cancer |
| Maastricht University | Netherlands | University Hospital/Cancer centre | Hepato-Pancreato-Biliary cancers |
| University of Leeds | U.K. | University Hospital/Cancer centre | Metastatic prostate cancer |
| The University of Edinburgh | U.K. | University Hospital/Cancer centre | Metastatic breast cancer |
| Fondazione IRCCS Istituto Nazionale dei Tumori | Italy | University Hospital/Cancer centre | (Advanced) lung cancer |
| Vrije Universiteit Brussel | Belgium | University Hospital/Cancer centre | (Advanced) lung cancer |
| Hospice Casa Sperantei | Romania | Specialist palliative care unit | Palliative care |
| Copenhagen University Hospital | Denmark | University Hospital/Palliative care unit | Palliative care |
